# Degradation of 1-alkyl-3-methylimidazolium tetrafluoroborate in an ultrasonic zero-valent zinc and activated carbon micro-electrolysis system

**DOI:** 10.1038/s41598-023-28237-4

**Published:** 2023-02-02

**Authors:** Ping Lyu, Wan Guo, Hang Qi, Xiang Yuan, Jinqi Ma, Xingmin Xu, Haimei Zhou

**Affiliations:** Faculty of Forensic Medicine, Henan University of Science and Technology, Luoyang, China

**Keywords:** Ionic liquids, Pollution remediation

## Abstract

Increased attention has been given to the removal of ionic liquids (ILs) from natural water environments. In this work, 5 kinds of 1-alkyl-3-methylimidazoliumtetrafluoroborate ([C_n_mim][BF_4_] (n = 2, 4, 6, 8, 10)) ILs were degraded in an ultrasonic zero-valent zinc (ZVZ) and activated carbon (AC) micro-electrolysis system. Optimization of degradation conditions and the degradation levels were studied by high performance liquid chromatography, the surface morphology of the ZVZ and AC changed before and after the reaction were observed by scanning electron microscope. The degradation intermediates were detected by gas chromatography- mass spectrometry and ion chromatography, and inferred the degradation pathway. The degradation effect of [C_4_mim][BF_4_] was best with ultrasonic assistance, pH 3 and an AC/ZVZ ratio of 1:1. The degradation of [C_n_mim][BF_4_] in aqueous solution exceeded 91.7% in 120 min, and the mineralization level exceeded 88.9%. The surface of smooth and dense ZVZ particles became loose flocculent and the porous surface of AC became larger and rougher after reaction. The degradation pathway suggested that the imidazolium ring was sulfurized or oxidized, and then the ring was opened to form N-alkyl formamide and N-methyl formamide. ZVZ/AC micro-electrolysis combined with ultrasonic irradiation is an effective method to remove ILs, which provides new insight into IL degradation.

## Introduction

Ionic liquids (ILs) have unique physical and chemical properties, which make them widely applicable in synthetic chemistry, electrochemistry, pharmacy, biology, nanotechnology, separation technology and other fields^1–8^. An increased number of ILs is used as media and solvents in cutting-edge science and technology. However, because of the low vapor pressure, good water solubility and stability of ILs, waste liquid that is discharged during the use of ILs remain in the soil or water body for a long time, which results in different extents of environmental pollution^9^. The potential toxicity of ILs has attracted attention, including the toxicity of ILs to higher plants, and the toxic effects on aquatic organisms, microorganisms, cells and skin^10,11^. Therefore, it is necessary to find a suitable and effective method to remove ILs from waste liquid.

Because most ILs are difficult to biodegrade, the chemical degradation of ILs has been discussed^12^, including Fenton oxidation degradation of ILs^13,14^, ultraviolet (UV)-H_2_O_2_ or UV-TiO_2_ photo-assisted degradation^15–19^, ultrasonic (US)-H_2_O_2_/acetic acid system oxidative degradation^20^, electrochemical oxidation and electro-Fenton system degradation^21–25^. Among the degradation systems reported above, some are time-consuming and inefficient, some are complex in subsequent treatment (such as the homogeneous Fenton-like system, which easily produces a large amount of iron-containing sludge, and the subsequent treatment cost is high), whereas others have a high energy consumption.

Micro-electrolysis technology is considered to require low energy consumption and is a high-efficiency wastewater treatment method. Previous studies have shown that zero-valent iron activated carbon (ZVI/AC) micro-electrolysis can degrade ILs^26,27^. On this foundation, zero valent zinc (ZVZ) was chosen to replace zero valent iron, because the potential of zinc was lower than that of iron, so it was speculated that the galvanic effect was stronger.

In this study, ZVZ and activated carbon (AC) were combined to form a micro-electrolysis system. The potential difference between low-potential zinc and high-potential carbon is large, and the IL wastewater to be degraded acts as an electrolyte to form numerous microgalvanic cells, in which zinc is the anode and carbon is the cathode and produces nascent hydrogen, Zn^2+^ and free radicals. The IL structure is destroyed by redox reaction. In this work, 1-alkyl-3-methylimidazolium tetrafluoroborate ([C_n_mim][BF_4_] (n = 2, 4, 6, 8, 10)) IL was degraded by a battery reaction without a power supply, which provided a scientific basis for the treatment of IL wastewater.

It was worth mentioning that the degradation of ILs in the micro-electrolysis system was related to their electrochemical stability, the results of this paper will provide relevant reference for the design and their electrochemical stability of ILs.

## Results

### Optimization of operational parameters of [C_4_mim][BF_4_] degradation in US-ZVZ/AC system

[C_4_mim][BF_4_] (1 mmol/L) was used as a model imidazolium IL to optimize the degradation of the imidazolium ILs at 30 °C in the US-ZVZ/AC micro-electrolysis system, for the initial pH, dosages and ratio of ZVZ and AC.

#### Effect of pH on [C_4_mim][BF_4_] degradation

**Figure 1:**
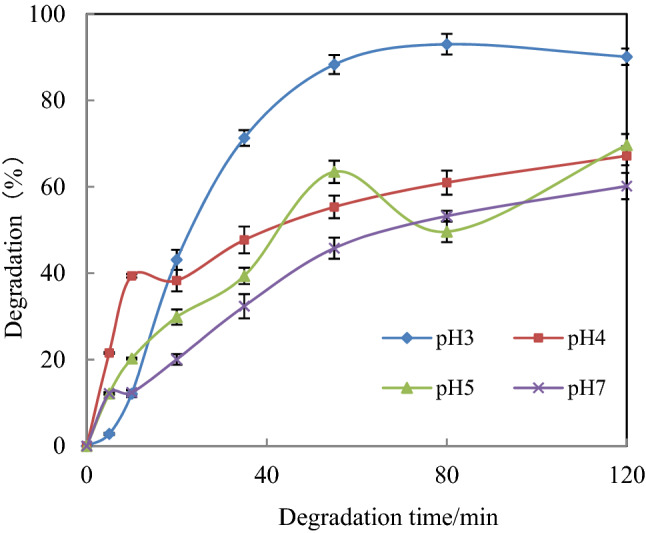
Effect of pH on [C_4_mim][BF_4_] degradation. ([[C_4_mim][BF_4_]] = 1 mmol/L, [AC] = 0.15 g, [ZVZ] = 0.15 g and ultrasound frequency = 45 kHz at 30 °C).

The influence of initial solution pH on the degradation of [C_4_mim][BF_4_] was investigated at four different pHs of 3.0, 4.0, 5.0 and 7.0 in the US-ZVZ/AC system. The degradation of [C_4_mim][BF_4_] was carried out by using a solution of 25 ml [C_4_mim][BF_4_] (1 mmol/L) 0.15 g AC and 0.15 g zinc powder, US 45 kHz, and the data are presented in Fig. 1. The degradation of [C_4_mim][BF_4_] increased with a decrease of solution pH, and the most efficient pH was 3.0. Under acidic condition, zinc powder has high activity in the free H^+^ environment and is easy to become an ionic state, thus accelerating the reaction. This result is analogous to our previous reports on imidazolium ILs in the US-ZVI/AC system^27^.

#### Effect of AC and ZVZ dosages on [C_4_mim][BF_4_] degradation

**Figure 2:**
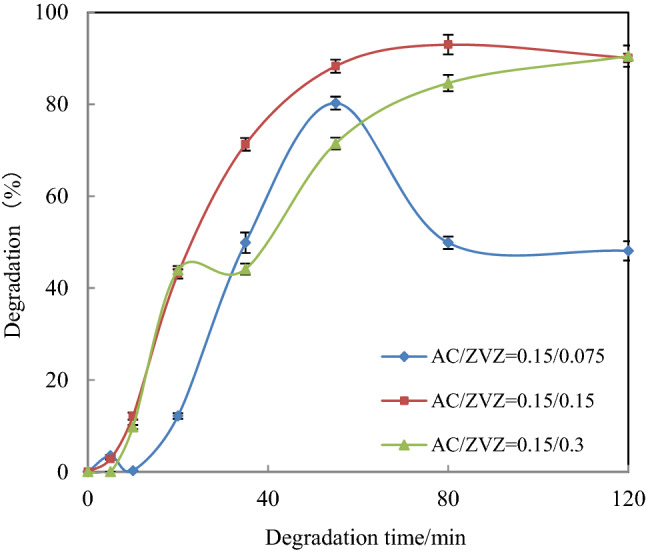
Effect of ratios of AC and ZVZ on [C_4_mim][BF_4_] degradation. ([[C_4_mim][BF_4_]] = 1 mmol/L, pH = 3.0 and ultrasound frequency = 45 kHz at 30 °C).

According to the method in “Degradation experiments”, the effect of AC/ZVZ ratio on the degradation efficiency of [C_4_mim][BF_4_] was examined at pH 3.0 and the three initial concentration ratios of AC/ZVZ were chosen as 1:2, 1:1 and 2:1, i.e., 0.15/0.3, 0.15/0.15 and 0.15/0.075 (g/g). The degradation effect of [C_4_mim][BF_4_] was best when the AC/ZVZ ratio is 1:1, as shown in Fig. 2.

**Figure 3:**
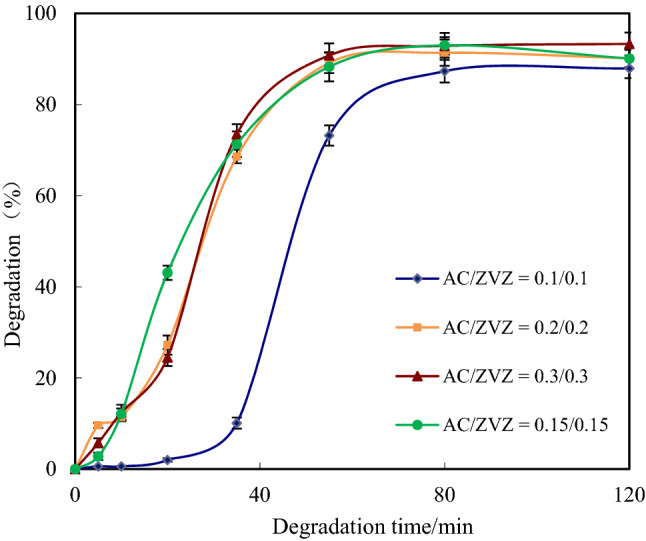
Effect of dosages of ZVZ and AC on [C_4_mim][BF_4_] degradation. ([[C_4_mim][BF_4_]] = 1 mmol/L, pH = 3.0 and ultrasound frequency = 45 kHz at 30 °C).

According to the above experimental method, four 25 ml 1 mmol/L [C_4_mim][BF_4_] aqueous solution systems were prepared, the pH was adjusted to 3, and the degradation of [C_4_mim][BF_4_] was determined by using 0.1 g/0.1 g, 0.15 g/0.15 g, 0.2 g/0.2 g and 0.3 g/0.3 g AC/ZVZ, respectively. The results are shown in Fig. 3. The degradation of 0.15 g/0.15 g AC/ZVZ was equivalent to that of 0.2 g/0.2 g or 0.3 g/0.3 g of AC/ZVZ, which was significantly better than that of 0.1 g/0.1 g of AC/ZVZ. The increase in AC and ZVZ increased the ionic zinc in the reaction system, which accelerated the reaction. Economically, a 0.15 g/0.15 g dosage was best in this experiment.

#### Effect of US irradiation on [C_4_mim][BF_4_] degradation

**Figure 4:**
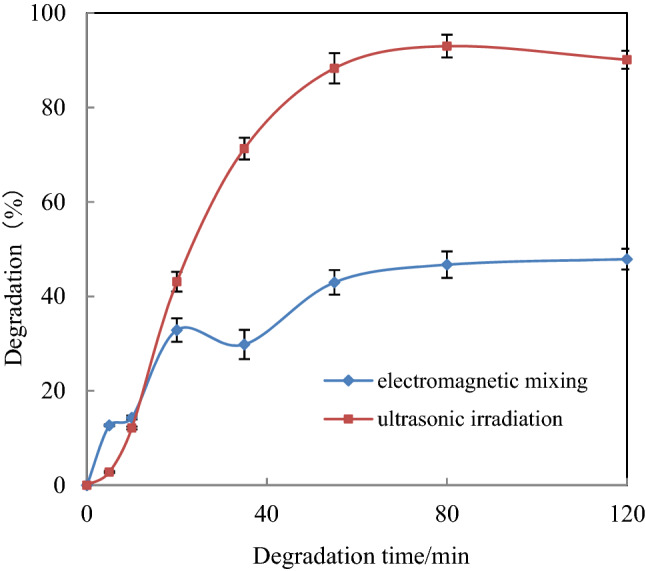
Effect of US irradiation on [C_4_mim][BF_4_] degradation. ([[C_4_mim][BF_4_]] = 1 mmol/L, [AC] = 0.15 g, [ZVZ] = 0.15 g, pH = 3.0 at 30 °C).

Based on the results from the experiments, the degradation of [C_4_mim][BF_4_] with magnetic stirring and ultrasound was discussed. Two 1 mmol/L [C_4_mim][BF_4_] systems were prepared, and the degradation was carried out under the above conditions with the assistance of 45 kHz ultrasound and magnetic stirring, respectively. Figure 4 shows that the degradation of [C_4_mim][BF_4_] can be increased significantly by using US assistance. The degradation extent of [C_4_mim][BF_4_] was 47.89% in 120 min and 93% under US-assisted conditions. This outcome may have resulted because of the cavitation effect of ultrasound. Many small bubbles are produced in the reaction liquid and these bubbles move, grow or burst with the vibration of the surrounding medium, which results in a local instantaneous high temperature and high pressure and a chemical effect. In air, water molecules decompose to produce highly active free radicals, such as H⋅, HO⋅ and O⋅, which can decompose highly stable organic compounds^20^. The mechanical and thermal effects of sound waves on the particle surface contribute to organic matter removal. In the ZVZ/AC micro-electrolysis system, many microgalvanic cells were generated between C and Zn, Zn (II), nascent hydrogen was released, and new free radicals were generated continuously under the action of ultrasound, which results in the effective decomposition of [C_4_mim][BF_4_] IL.

In our experiments, no degradation of [C_4_mim][BF_4_] occurred under 45 kHz US radiation, whereas the combination of US-ZVZ degraded ~ 13% of the [C_4_mim][BF_4_] at 120 min. The degradation efficiency in the US-ZVZ/AC system improved significantly, so a synergistic effect occurred between the US and ZVZ/AC system, which improves the degradation of [C_4_mim][BF_4_].

#### Effect of different parameters on [C_4_mim][BF_4_] degradation

As can be seen from Table 1, the optimal conditions for [C_4_mim][BF_4_] degradation were pH3, AC/ZVZ dosage of 0.15 g/0.15 g (ratio 1:1) and ultrasound frequency 45 kHz ([IL] = 1 mmol/L at 30 °C).

**Table 1:** Results of [C_4_mim][BF_4_] degradation in US-ZVZ/AC system under the different conditions.

pH	AC/ZVZ ratio	AC/ZVZ dosage	US	(Degradation rate)_max_ within 120 min
3	1:1	0.15 g/0.15 g	45 kHz	93.00
4	1:1	0.15 g/0.15 g	45 kHz	67.17
5	1:1	0.15 g/0.15 g	45 kHz	69.66
6	1:1	0.15 g/0.15 g	45 kHz	60.17
3	2:1	0.15 g/0.075 g	45 kHz	49.87
3	1:1	0.15 g/0.15 g	45 kHz	93.00
3	1:2	0.15 g/0.3 g	45 kHz	90.49
3	1:1	0.1 g/0.1 g	45 kHz	87.93
3	1:1	0.2 g/0.2 g	45 kHz	91.37
3	1:1	0.3 g/0.3 g	45 kHz	93.31
3	1:1	0.15 g/0.15 g	45 kHz	93.00
3	1:1	0.15 g/0.15 g	45 kHz	93.00
3	1:1	0.15 g/0.15 g	Without US	47.89

[C_4_mim][BF_4_] concentration: 1 mmol/L, Degradation temperature: 30 °C.

AC used in the experiments had been saturated by IL in advance, and the concentration falling of IL in the degradation process was no longer affected by AC adsorption^27^.

It is worth noting that under some degradation conditions, the degradation level of ionic liquid has a low point in the early stage of degradation, most of which are at 10 min. It is speculated that the degradation kinetics of different reaction stages are different. The kinetics of this degradation reaction needs to be further discussed.

### Surface characterization of ZVZ and AC before and after degradation

**Figure 5:**
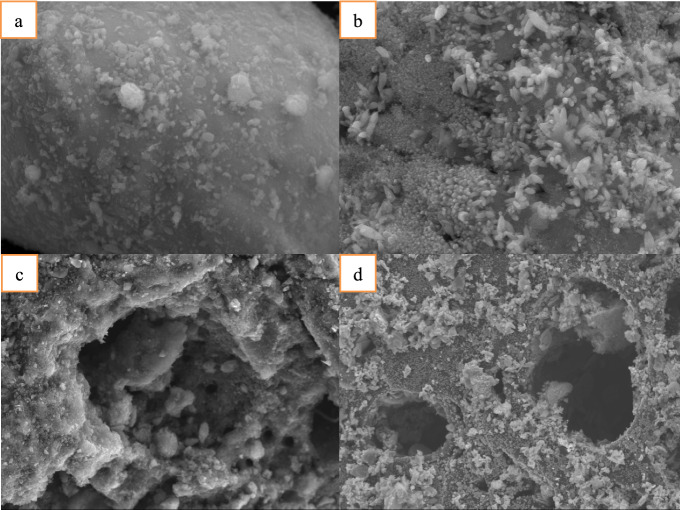
Surface characterization of ZVZ and AC before and after [C_4_mim][BF_4_] degradation.

SEM images (Fig. 5) show obvious differences between the ZVZ and AC surface before and after the reaction. The surface of the initial ZVZ particles was smooth and dense (Fig. 5a). After 120 min of reaction, most particles formed a loose flocculent structure and were covered by a scale of clusters (Fig. 5b), which means that a zinc-oxide layer formed on the ZVZ surface during the reaction. Granular AC has an irregular porous structure, as shown in Fig. 5c. Some ILs are adsorbed in the pores (AC should be saturated with [C_4_mim][BF_4_] in advance if necessary). After US-assisted micro-electrolysis, the ILs that had adsorbed in the pores disappeared, the AC pores were larger and the surface was rough (Fig. 5d). The surface morphology of ZVZ and AC changed visibly before and after the reaction, and the US assistance promoted mass transfer on the AC surface.

### Degradation of imidazolium ILs with different alkyl chain lengths

**Figure 6:**
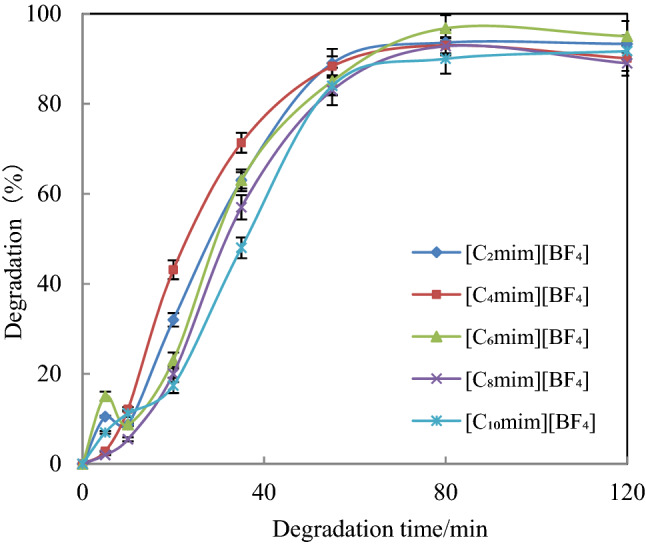
Degradation of imidazolium ILs with different alkyl chain lengths. ([IL] = 1 mmol/L, [AC] = 0.15 g, [ZVZ] = 0.15 g, pH = 3.0 and ultrasound frequency = 45 kHz at 30 °C).

Degradation was carried out on 1 mmol/L [C_2_mim][BF_4_], [C_4_mim][BF_4_], [C_6_mim][BF_4_], [C_8_mim][BF_4_], [C_10_mim][BF_4_] aqueous solution (pH 3) under optimized conditions according to the method described in section “Degradation experiments”. HPLC analysis was carried out according to the method described in “Analytical procedures”, and the degradation of imidazolium ILs with different side chains was observed. Figure 6 shows that the extent of imidazolium IL degradation with different alkyl side chains was slightly different. The degradation of [C_2_mim][BF_4_], [C_4_mim][BF_4_], [C_6_mim][BF_4_], [C_8_mim][BF_4_] and [C_10_mim][BF_4_] was 93.6%, 93.0%, 96.8%, 92.8% and 91.7%, respectively, at 120 min. No significant influence of alkyl side chain length was observed, and this result agrees with that for iron carbon micro-electrolysis IL degradation by our team^26^.

### Analysis of degradation intermediates and pathway for [C_n_mim][BF_4_]

#### UV spectra changes during [C_4_mim][BF_4_] degradation

**Figure 7:**
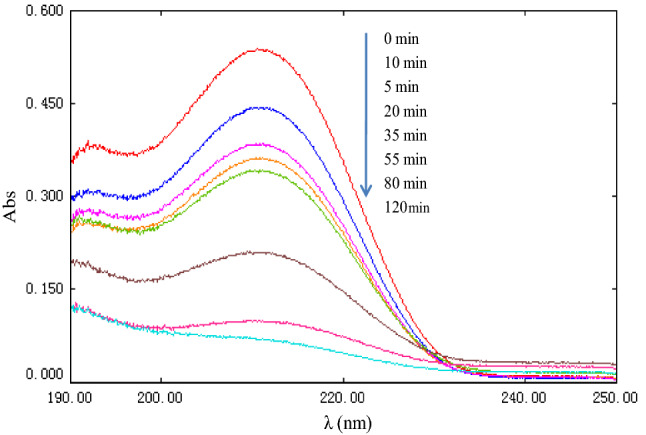
UV spectra for degradation reaction mixtures of [C_4_mim][BF_4_] at various reaction times in US-assisted ZVZ/AC system: [[C_4_mim][BF_4_]] = 1 mmol/L, [AC] = 0.15 g, [ZVZ] = 0.15 g, pH = 3.0 and ultrasound frequency = 45 kHz at 30 °C.

To clarify the changes in structural characteristics of [C_n_mim][BF_4_] in the ZVZ/AC micro-electrolysis system assisted by US irradiation, representative UV spectra for the solution were determined as a function of reaction time. Figure 7 shows that the absorption spectrum of [C_4_mim][BF_4_] in water was characterized by a main band in the ultraviolet region at 212 nm. This peak was associated with the imidazolium ring of [C_4_mim][BF_4_]. With an increase in degradation time, the absorption of the degradation mixture at 212 nm decreased gradually. The absorption peak at 212 nm almost disappeared at 120 min, which indicates that the opening of the imidazolium ring and effective degradation of [C_4_mim][BF_4_]. [C_2_mim][BF_4_], [C_6_mim][BF_4_], [C_8_mim][BF_4_] and [C_10_mim][BF_4_] have similar trends.

#### Degradation intermediates for [C_n_mim][BF_4_]

The identification of intermediates in the degradation process can provide valuable information about the degradation pathway. Therefore, we took 10 ml of [C_n_mim][BF_4_] degradation solution at different reaction times (20, 40, 70 and 120 min), saturated the sample with sodium chloride, extracted the intermediate products with organic solvent, and analyzed and identified the product by GC–MS. The inorganic anions that were produced by degradation were detected by ion chromatography. The chemical structures and names of the intermediates are shown in Table 2.

**Table 2:** Degradation intermediates of [C_n_mim][BF_4_] detected by GC–MS and IC.

IL	Intermediate	Retention time (min)	Main fragment (m/z)	Molecular weight
[C_2_mim][BF_4_]	***e***_***2***_	10.16	58	130
	***i***	4.02	59	59
	***g***_***2***_	4.68	73	73
	***j*** NO_3_^–^			
[C_4_mim][BF_4_]	***d***_***4***_	12.55	58,30,186	186
	***i***	3.99	59,30	59
	***g***_***4***_	7.17	58,30,101	101
	***j*** NO_3_^–^			
[C_6_mim][BF_4_]	***a***_***6***_	17.50	165,198	198
	***b***_***6***_	14.38	98,182	182
	***c***_***6***_	15.27	56,142	212
	***d***_***6***_	14.89	58	214
	***f***_***6***_	10.42	58	157
	***g***_***6***_	10.18	58	129
	***j*** NO_3_^–^			
[C_8_mim][BF_4_]	***a***_***8***_	19.59	193,226	226
	***b***_***8***_	16.60	98,210	210
	***c***_***8***_	17.47	142	240
	***d***_***8***_	17.11	60	242
	***f***_***8***_	13.09	74	185
	***g***_***8***_	12.86	58	157
	***j*** NO_3_^–^			
[C_10_mim][BF_4_]	***a***_***10***_	21.58	221,254	254
	***b***_***10***_	18.76	98,238	238
	***c***_***10***_	19.50	142	268
	***d***_***10***_	19.16	60	270
	***f***_***10***_	15.51	74	213
	***g***_***10***_	15.31	59	185
	***h***_***10***_	11.72	99	183
	***j*** NO_3_^–^			

Four degradation products were detected in [C_2_mim][BF_4_] and [C_4_mim][BF_4_]. ***e***_***2***_ in the table is the urea that was formed by the oxidation of three C-H on the [C_2_mim][BF_4_] imidazole ring to three carbonyl groups and the opening of the imidazole ring. ***g***_***2***_ and ***i*** are products that were formed by bond breakage between the two carbonyl groups of ***e***_***2***_. Seven, seven and eight degradation products were detected in [C_6_mim][BF_4_], [C_8_mim][BF_4_] and [C_10_mim][BF_4_], respectively. For [C_10_mim][BF_4_] as an example, where ***a***_***10***_ is obtained by sulfurizing the H of C2 on the imidazolium ring, ***b***_***10***_ is the product of oxidation of C-H on C2 of the imidazole ring to carbonyl, ***c***_***10***_ is the product of oxidation of C-H on the other two C of the imidazole ring to carbonyl, ***d***_***10***_ is the urea that formed after the opening of the ***c***_***10***_ imidazole ring, ***f***_***10***_ is the product of ***d***_***10***_ removing N-methylformamide, ***g***_***10***_ is the product of further decomposition of ***d***_***10***_ and ***f***_***10***_, and ***h***_***10***_ is generated after dehydrogenation.

#### Degradation pathway for [C_n_mim][BF_4_]

According to the chemical structure of the intermediates, we propose a degradation pathway of [C_2_mim][BF_4_] in which oxidant radicals that are generated in the US-ZVZ/AC reaction system attack H on the 2, 4, 5 carbon atom of the imidazolium ring to produce 1-ethyl-3-methyl-2, 4, 5-trioxyimidazole, and then oxidize to open the ring to obtain ***e***_***2***_. ***g***_***2***_ and ***i*** are obtained by C–C bond breakage between the two carbonyls.

The degradation products of [C_4_mim][BF_4_] are consistent with those of [C_2_mim][BF_4_], so it is speculated that [C_4_mim][BF_4_] has a similar degradation pathway.

The possible degradation pathways of [C_6_mim][BF_4_], [C_8_mim][BF_4_] and [C_10_mim][BF_4_] ILs in US-ZVZ/AC system are as follows:

(i) H on the imidazole ring is sulfurized to form 1-alkyl-3-methyl-2-thioimidazole, or oxidized to form 1-alkyl-3-methyl-2-oxyimidazole, and further oxidized to give 1-alkyl-3-methyl-2, 4, 5-trioxyimidazole; (ii) ring opening of imidazole, bond breakage between two carbonyl groups to form corresponding intermediate products, and decomposition to form N-alkyl formamide and N-methyl formamide; (iii) degradation of alkyl formamide to form alkyl chain fatty acids and nitrate ions.

**Figure 8:**
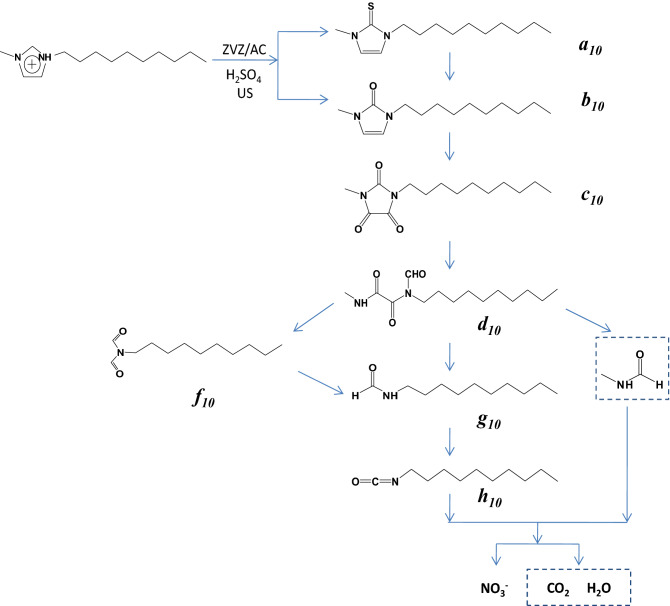
Degradation pathway suggested for [C_10_mim]^+^.

The possible degradation pathway of [C_10_mim][BF_4_] is shown in Fig. 8.

In the literature, few studies exist on the degradation products of imidazolium ILs. Our results are inconsistent with those reported^28–30^ because the degradation methods, products and degradation pathways are different.

In addition, in the micro electrolysis system, there might be chemical decomposition of IL at the same time. This paper only speculates the degradation path based on the detected degradation products.

### Mineralization of imidazolium ILs

**Figure 9:**
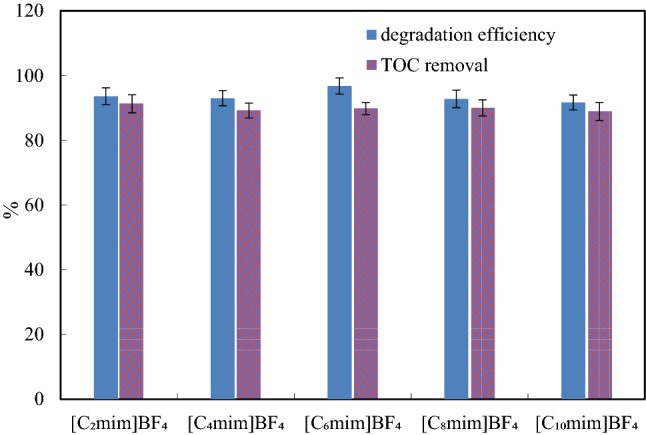
TOC removal of [C_n_mim][BF_4_] (1 mmol/L) in US-ZVZ/AC system: [AC] = 0.15 g, [ZVZ] = 0.15 g, pH = 3.0 and ultrasound frequency = 45 kHz at 30 °C.

The mineralization of imidazolium IL [C_n_mim][BF_4_] (n = 2, 4, 6, 8, 10) can be evaluated by the TOC content. The TOC before and after [C_n_mim][BF_4_] degradation can be determined, and the TOC removal during degradation can be calculated. We measured the TOC of the ILs degradation mixture at 0 and 120 min, and obtained the TOC removal at 120 min (Fig. 9). That of [C_2_mim][BF_4_], [C_4_mim][BF_4_], [C_6_mim][BF_4_], [C_8_mim][BF_4_] and [C_10_mim][BF_4_] was 91.3%, 89.2%, 89.8%, 90.0% and 88.9%, respectively, that is, the TOC removal ranged from 88.9 to 91.3%. Therefore, more than 88% of the TOC could be removed during 120 min of the degradation reaction.

According to the degradation pathway of [C_n_mim][BF_4_] IL proposed above, the detected products, such as N-alkyl formamide and N-methyl formamide, may have been further mineralized. The TOC removal of the degradation system was high, which shows that the degradation was effective. These data are higher than the TOC removal rate of IL degradation reported in the literature^13,25,26^.

The experimental results show that the removal of TOC at 120 min was lower than the degradation level at the same time, which indicates that in addition to a small amount of parent compound, the degradation byproducts also coexist in the degradation solution, and there is insufficient time to further decompose and mineralize at 120 min. The TOC measurements show that [C_n_mim][BF_4_] can be decomposed rapidly and mineralized under US-ZVZ/AC micro-electrolysis.

## Conclusion

This study demonstrated that 1-alkyl-3-methylimidazolium IL can be degraded rapidly and effectively in the US-ZVZ/AC micro-electrolysis system, and the degradation exceeded 91.7% in 120 min. The degradation of imidazolium IL [C_n_mim][BF_4_] (n = 2, 4, 6, 8, 10) with different side chain lengths was almost unaffected by the length of the alkyl side chain. During the degradation of 1-alkyl-3-methylimidazolium tetrafluoroborate, H on the imidazolium ring was sulfurized to 1-alkyl-3-methyl-2-thioimidazole, or oxidized to 1-alkyl-3-methyl-2-oxyimidazole, and further oxidized to 1-alkyl-3-methyl-2, 4, 5-trioxyimidazole. Through the ring opening of imidazole and bond breakage between two carbonyl groups, corresponding intermediate products formed and decomposed into N-alkyl formamide and N-methyl formamide, and then alkyl chain fatty acids (such as formic acid) and nitrate ions were generated. More than 88.9% of the TOC was removed during the degradation.

Compared with zero-valent iron activated carbon (ZVI/AC) micro-electrolysis^26^, the degradation rate of ionic liquid under zero-valent zinc activated carbon (ZVZ/AC) micro-electrolysis system was slightly faster, and the removal level of TOC was higher. It may be that the potential difference of ZVZ/AC was larger than that of ZVI/AC, and more active species were produced in the ZVZ/AC system. The degradation pathways of the two were similar, and the degradation products detected in ZVZ/AC micro-electrolysis were more abundant.

ZVZ/AC micro-electrolysis technology is low cost and easy to operate. The high degradation level and TOC removal indicate that the US-ZVZ/AC system is effective and environmentally friendly in the removal of 1-alkyl-3-methylimidazolium tetrafluoroborate ILs. The results of this work have important guiding significance to eliminate the environmental impact of IL waste and evaluate the environmental behavior of common ILs.

## Methods

### Chemicals

ILs were from Chengjie Chem. Co. Ltd. (Shanghai, China; purity > 99%) and were used without further purification. The ILs that were used in our work included: 1-ethyl-3-methylimidazolium tetrafluoroborate ([C_2_mim][BF_4_]), 1-butyl-3-methylimidazolium tetrafluoroborate ([C_4_mim][BF_4_]), 1-hexyl-3-methylimidazolium tetrafluoroborate ([C_6_mim][BF_4_]), 1-octyl-3-methylimidazolium tetrafluoroborate ([C_8_mim][BF_4_]) and 1-decyl-3-methylimidazolium tetrafluoroborate ([C_10_mim][BF_4_]). Stock solutions of [C_n_mim][BF_4_] (n = 2, 4, 6, 8, 10) (25 mmol/L) were prepared in purified water (Milli-Q, Millipore, Co., USA) and stored at 4 °C. The names, molecular masses and chemical structures of the ILs are listed in Table 3.

**Table 3:** Names, molecular mass and chemical structures of ILs studied.

Name	Abbreviation	Molecular Mass	Chemical formula
1-ethyl-3-methylimidazolium tetrafluoroborate	[C_2_mim][BF_4_]	197.97	
1-butyl-3-methylimidazolium tetrafluoroborate	[C_4_mim][BF_4_]	226.03	
1-hexyl-3-methylimidazolium tetrafluoroborate	[C_6_mim][BF_4_]	254.08	
1-octyl-3-methylimidazolium tetrafluoroborate	[C_8_mim][BF_4_]	282.13	
1-decyl-3-methylimidazolium tetrafluoroborate	[C_10_mim][BF_4_]	310.18	

ZVZ powders and AC (35–50 mesh) were from Beijing Chem. Co., Ltd. Ammonium formate, ammonium acetate, ammonium propionate, sodium nitrate, sodium sulfate, sodium chloride and sodium tetrafluoroborate (Guaranteed reagent GR) were provided by Shandong Xiya Chemical Co., Ltd. (Shandong, China) and Tianjin Guangfu Technology Development Co., Ltd. (Tianjin, China).

Acetonitrile and methanol (LC-grade) were from Kermal Chemical Reagents Development Center (Tianjin, China). Other reagents, such as ethyl acetate, ether, benzene, triethylamine, H_3_PO_4_ and KH_2_PO_4_, were of analytical grade from Beijing Chemical Factory (Beijing, China).

### Degradation experiments

IL degradation experiments were conducted in a flask (50 mL) in an ultrasonic cleaner (300 W, 45 kHz or 80 kHz, KQ-300GVDV, Kunshan Ultrasonic Instrument Co. Ltd., China) with a temperature-controlled container filled with water. The experimental operation and treatment of AC was described in the literature^27^. The degradation reaction was initiated by the addition of a given amount of zinc powder. Samples were collected at various degradation times. The degradation was stopped at 120 min. All experiments were repeated and the reaction temperature was kept at 30 °C. The pH of the initial solution was adjusted with 10% sulfuric acid.

### Analytical procedures

#### High performance liquid chromatography (HPLC) analysis

HPLC (Waters Series 1525, USA) was used to determine the concentration of the ILs as described in literature^26^. The elution profiles were monitored at 212 nm. A mixture of methanol (35%, v/v) with 25 mmol/L phosphate buffer (KH_2_PO_4_/H_3_PO_4_) in 0.5% triethylamine at pH 3 was used as the mobile phase for the analysis of [C_n_mim][BF_4_](n = 2, 4, 6). A mixture of acetonitrile (35%, v/v) with 25 mmol/L of phosphate buffer (KH_2_PO_4_/H_3_PO_4_) in 0.5% triethylamine (pH3.0) was used for the analysis of [C_8_mim][BF_4_] and [C_10_mim][BF_4_]. Each sample was measured in triplicate at various reaction times. According to the measured concentrations of [C_n_mim][BF_4_] at the 0 degradation time, the degradation degree of [C_n_mim][BF_4_] at moment t can be calculated.

#### Scanning electron microscope (SEM) analysis

SEM (JEOLJSM-7800F, Japan) was used for the analysis of the ZVZ and AC particle surface morphology before and after the degradation reaction at a 20-kV and 5-kV beam potential.

#### Gas chromatography–mass spectrometry (GC–MS) analysis

GC–MS (Agilent 6890/5973N series, USA) was used to identify the degradation intermediates. The degradation products were extracted with a mixture of benzene-ethyl acetate-ether (2:2:1) and analyzed as described in the literature^24^. The structural identification of intermediates was based on the NIST05 library for the mass spectra and combined with an interpretation of the fragmentation pathways.

#### Ion chromatography (IC) analysis

IC (Thermo Fisher ICS600, USA) was used to analyze the anionic species in solution after degradation. The analytical IC conditions were: column: IonPac AS14 (4.0 × 250 mm) + AG14, (4.0 × 50 mm); mobile phase: 3.5 mmol/L Na_2_CO_3_—1.0 mmol/L NaHCO_3_ aqueous solution; column temperature: 30 °C; suppressor current: 24 mA; flow rate: 1.2 mL/min; injection volume: 10 μL; and a Chromeleon7 workstation was used for data processing.

#### Total organic carbon (TOC) analysis

The TOC of the initial and degraded samples was measured by a TC/TN analyzer (Analytik Jena, multi N/C 2100, Germany) according to a thermal catalytic oxidation principle.

## Data Availability

Some or all data that support the findings of this study are available from the corresponding author upon reasonable request.
